# Understanding and Treating Nightmares: A Comprehensive Review of Psychosocial Strategies for Adults and Children

**DOI:** 10.7759/cureus.70044

**Published:** 2024-09-23

**Authors:** Namita Sahu, Pradeep S Patil, Asmita -, Imyarila Longkumer

**Affiliations:** 1 Psychiatry, Jawaharlal Nehru Medical College, Datta Meghe Institute of Higher Education & Research, Wardha, IND

**Keywords:** cognitive behavioral therapy (cbt), imagery rehearsal therapy (irt), mental health, nightmares, psychosocial interventions, sleep disturbances

## Abstract

Nightmares are distressing dreams that evoke strong negative emotions, such as fear or anxiety, often leading to waking from sleep and subsequent sleep disruption. They are prevalent across various age groups, with significant psychological and physiological health implications. This review explores the nature of nightmares, distinguishing them from other sleep disturbances like night terrors and sleep paralysis. It examines the prevalence of nightmares in children and adults, highlighting their impact on mental health and daily functioning. The review also emphasizes the importance of addressing nightmares through effective treatment strategies.

While pharmacological options are available, psychosocial interventions offer promising non-pharmacological solutions. Cognitive behavioral therapy (CBT), imagery rehearsal therapy (IRT), and mindfulness-based therapies are discussed as key approaches for managing nightmares. These strategies focus on altering maladaptive thought patterns and emotional responses, reducing nightmares' frequency and intensity, and improving overall sleep quality. Psychosocial interventions provide a comprehensive approach to treating nightmares by addressing the underlying cognitive and emotional factors, benefitting individuals across different age groups. This review aims to highlight the efficacy of these strategies and their role in enhancing the quality of life for those affected by persistent nightmares.

## Introduction and background

Nightmares are distressing dreams that often threaten an individual’s safety, security, or physical integrity, leading to a sudden awakening [1]. Unlike typical dreams, nightmares are characterized by intense negative emotions such as fear, anxiety, or sadness, which can linger even after the dreamer has woken up [2]. These disturbing experiences occur predominantly during the rapid eye movement (REM) stage of sleep, a phase characterized by vivid dreaming. Upon waking from a nightmare, the person can usually recall the content in vivid detail, which often contributes to ongoing distress and can disrupt their sleep pattern [3].

It is important to distinguish nightmares from other sleep disturbances, particularly night terrors. While nightmares occur during REM sleep and involve detailed recollection of the dream upon waking, night terrors are a type of parasomnia that occurs during non-REM sleep, especially in the deep stages [4]. Individuals experiencing night terrors may scream, thrash, or exhibit signs of panic, but they typically do not fully awaken or remember the event the following day [1]. Other sleep disturbances, such as sleep paralysis and REM sleep behavior disorder (RBD), may also be confused with nightmares. Sleep paralysis involves a sensation of being awake but unable to move, often accompanied by feelings of fear or a perceived threat. In contrast, RBD involves physically acting out dreams, sometimes violently, though these do not necessarily involve the emotional terror that characterizes nightmares [1].

Nightmares are a common phenomenon across various age groups, though their prevalence can vary. In children, nightmares are particularly frequent, with studies suggesting that approximately 10-50% experience them regularly, especially during the ages of three to six years [5]. As children grow older, the frequency of nightmares typically decreases. In adults, the prevalence is lower but still significant, with about 2-8% of the population experiencing frequent nightmares. However, certain populations, such as those suffering from post-traumatic stress disorder (PTSD) or other anxiety disorders, may experience nightmares at much higher rates, often with greater severity [6].

The impact of nightmares extends far beyond the discomfort experienced during sleep. Frequent nightmares can lead to considerable psychological consequences, including heightened levels of anxiety, depression, and an increased risk of developing other mental health disorders [7]. Physiologically, nightmares can result in sleep fragmentation, leading to poor sleep quality and chronic sleep deprivation. This lack of restful sleep can impair daytime functioning, affecting concentration, memory, and emotional regulation. The cumulative effect of recurring nightmares can significantly diminish an individual's quality of life, underlining the importance of addressing and managing these sleep disturbances effectively [8].

Despite their prevalence, nightmares are often overlooked as a serious health concern. However, when nightmares become frequent or intense, they can cause substantial distress and significantly disrupt daily life [9]. Persistent nightmares not only exacerbate underlying psychological conditions, such as PTSD, but can also contribute to the development of new mental health issues. As a result, treating nightmares is crucial not just for reducing the frequency of these distressing episodes but also for improving the overall mental and emotional well-being of those affected [9].

While pharmacological treatments are available for nightmares, they often come with side effects and may not address the underlying causes. Psychosocial strategies, on the other hand, offer non-pharmacological interventions that can be highly effective in managing nightmares [10]. These strategies include cognitive behavioral therapy (CBT), imagery rehearsal therapy (IRT), and mindfulness-based therapies. Such approaches focus on altering the thought patterns and behaviors that contribute to nightmares, helping individuals regain control over their sleep, and reducing the frequency and intensity of these distressing events. By addressing both the cognitive and emotional aspects of nightmares, psychosocial interventions provide a holistic and effective approach to treatment, benefiting individuals across all age groups [10].

## Review

Etiology of nightmares

Nightmares are complex phenomena shaped by various psychological, biological, genetic, environmental, and social factors. Understanding these elements can offer valuable insights into their occurrence and potential treatment strategies [11]. Psychological factors play a crucial role in the development of nightmares, with stress, anxiety, and trauma being primary contributors. High levels of stress can lead to increased arousal during sleep, making individuals more vulnerable to nightmares. Stressful life events, such as job loss or relationship problems, often trigger intense dreams that mirror these anxieties [12]. Similarly, anxiety disorders, including generalized anxiety disorder and situational anxiety, frequently manifest in nightmares. Individuals may dream about their fears or worries, creating a cycle of anxiety and disrupted sleep. Trauma, especially from events like accidents, violence, or natural disasters, can result in recurrent nightmares that often replay aspects of the trauma, serving either as a coping mechanism or as a manifestation of unresolved emotions [13].

Mental health disorders also significantly impact the prevalence of nightmares. PTSD is particularly associated with vivid, distressing dreams that relive traumatic experiences, leading to significant sleep disruption. Individuals with PTSD often report recurrent nightmares that are emotionally intense and disturbing [14]. Additionally, depression is linked to changes in sleep architecture and increased REM sleep, both of which can contribute to a higher frequency of nightmares. Those suffering from depression may experience negative dream content that reflects their mood, further complicating their mental health [15]. Biological and genetic factors also play a role in the occurrence of nightmares. Research suggests that there may be a hereditary component to nightmares. Family studies indicate that individuals with a family history of nightmares or sleep disorders are more likely to experience them. Genetic predispositions may also increase susceptibility to anxiety and mood disorders, which are closely associated with nightmares [16].

The role of neurotransmitters and specific brain regions is critical in understanding nightmares. Imbalances in neurotransmitters such as serotonin and norepinephrine can affect sleep regulation and dream content [17]. These chemicals are essential for mood regulation and can influence the emotional tone of dreams. Additionally, the limbic system, particularly the amygdala, plays a key role in processing emotions and is highly active during REM sleep, potentially contributing to the emotional intensity of nightmares. Disruptions in the prefrontal cortex, responsible for rational thought and emotional regulation, can lead to less control over dream content, resulting in more vivid and distressing nightmares [17]. Environmental and social factors also significantly influence the occurrence of nightmares. Family dynamics can have a profound impact on both children and adults.

Family stressors, such as conflict or instability, can lead to nightmares as individuals internalize these issues, which manifest as anxiety in their dreams [18]. Additionally, exposure to violent or distressing media content can affect dream content, particularly in children, where graphic images or themes can seep into dreams, resulting in nightmares. Cultural beliefs and practices surrounding dreams can also shape how individuals interpret and respond to nightmares. In some cultures, nightmares may be viewed as omens or messages, influencing the psychological response to these experiences [19]. Major life changes or traumatic events can also trigger nightmares. Significant transitions, such as moving, starting a new job, or experiencing a loss, often bring about stress and anxiety, which can disrupt sleep patterns and contribute to the occurrence of nightmares. Traumatic events can leave lasting psychological impacts that manifest in dreams, leading to recurrent nightmares [20].

Nightmares in children

Nightmares are a common experience in children, with their prevalence peaking at various developmental stages. Approximately 50% of children aged 3 to 6 years frequently experience nightmares, while around 20% of children aged 6 to 12 years report the same. Compared to adults, children’s nightmares often involve more concrete, age-appropriate themes such as monsters, ghosts, or animals. The content of these nightmares typically reflects the developmental fears and anxieties common at these ages [21]. Frequent nightmares in children can have significant effects on their emotional well-being, behavior, and academic performance. Nightmares can lead to increased anxiety, mood disturbances, and difficulties with emotional regulation. Children who experience frequent nightmares are at higher risk for hyperactivity, attention problems, and poor academic performance. Recurring nightmares can also contribute to insomnia symptoms, such as difficulty falling asleep and staying asleep [22].

Several psychosocial interventions have proven effective in managing nightmares in children. CBT for children (CBT-C) includes techniques such as IRT, where children visualize and rehearse a less frightening ending to their nightmares during waking hours. This approach can reduce the frequency and intensity of nightmares. Cognitive restructuring, another CBT technique, helps children reframe their thoughts about nightmares and develop coping strategies, which can alleviate anxiety and improve sleep quality [23]. Parental guidance and support are crucial in managing nightmares in children. A consistent bedtime routine can help children feel more secure and reduce sleep-related anxiety. Creating a safe sleep environment by ensuring the child’s bedroom is comfortable and free from stressors can also help prevent nightmares.

Additionally, parents can offer emotional support by helping children process their bad dreams and discussing their fears and triggers in a calm, supportive setting [24]. Play therapy and creative expression techniques, such as art, storytelling, and play, can assist children in expressing and processing their fears and anxieties in a developmentally appropriate manner. For children experiencing frequent nightmares, particularly those related to trauma, professional counseling may be necessary. Although medication is generally not recommended for children, it may be prescribed in certain cases [25].

Nightmares in adults

Nightmares in adults are a significant concern due to their association with various mental health conditions, their impact on daily functioning, and the availability of psychosocial strategies for treatment. Understanding these aspects is crucial for effective management and support [26]. Nightmares are frequently linked to several mental health disorders, particularly PTSD, depression, and anxiety. PTSD is characterized by recurrent nightmares that often reflect traumatic experiences, exacerbating anxiety and distress. Individuals with PTSD may find themselves trapped in a cycle where the emotional turmoil from their nightmares heightens their symptoms, leading to further sleep disturbances. Additionally, adults with nightmare disorder often have co-occurring conditions such as depression and anxiety [27]. The distress caused by frequent nightmares can aggravate these mental health issues, creating a feedback loop where the conditions contribute to increased nightmare frequency and severity. Other psychiatric disorders, including borderline personality disorder and schizophrenia, have also been linked to nightmares, underscoring the complex interplay between sleep disturbances and mental health [28].

The impact of nightmares extends beyond mere sleep disruption, affecting various aspects of daily life. Frequent nightmares can lead to excessive daytime sleepiness, impairing concentration and productivity at work or school. This diminished focus can result in decreased performance and increased absenteeism, further compounding stress and anxiety. Moreover, the emotional distress caused by nightmares can strain personal relationships, leading to irritability or withdrawal and resulting in misunderstandings and conflicts with family and friends. Over time, the cumulative effects of nightmares can diminish the quality of life, characterized by increased anxiety, emotional dysregulation, and a general sense of distress. If left unaddressed, these issues can contribute to further mental health deterioration [9].

Fortunately, several effective psychosocial strategies are available for treating nightmares in adults. CBT for insomnia (CBT-I) is a structured program that addresses the thoughts and behaviors contributing to insomnia and nightmares. This therapy helps individuals develop healthier sleep habits and coping mechanisms, ultimately reducing the frequency and severity of nightmares. Another effective technique is IRT, where individuals visualize a positive ending to their nightmares while awake. This practice can help alter the emotional response to the nightmare and reduce its recurrence [29].

In addition to these therapies, mindfulness and relaxation techniques play a vital role in managing nightmares. Practices such as mindfulness meditation and relaxation exercises help reduce anxiety and improve overall sleep quality [30]. By promoting a sense of calm, these techniques make it easier for individuals to cope with the distress associated with nightmares. Furthermore, group therapy or support groups can provide emotional support and coping strategies. Connecting with others who experience similar issues fosters a sense of community and shared understanding, which can be beneficial for individuals dealing with nightmare-related distress [30]. A comparison of nightmares in children and adults, including prevalence, impact, and treatment approaches, is shown in Table 1.

**Table 1:** Comparison of nightmares in children and adults: prevalence, impact, and treatment approaches

Aspect	Nightmares in Children	Nightmares in Adults
Prevalence [31]	Common during early childhood, particularly between ages 3-6.	Less frequent compared to children but can increase in frequency with stress or trauma.
Content [32]	Often related to fears, monsters, or imagined threats.	More complex, often involving real-life concerns, stress, or trauma.
Frequency [33]	Higher in children, peaking during early childhood.	Less frequent, though, may increase during stress or mental health conditions.
Psychological Impact [14]	It can affect emotional regulation and cause fear or reluctance to sleep.	It can contribute to mental health conditions such as anxiety, depression, and post-traumatic stress disorder (PTSD).
Associated Conditions [14]	Often linked with normal developmental stages and temporary anxieties.	Commonly associated with PTSD, anxiety disorders, depression, or significant life stressors.
Treatment Strategies [34]	Cognitive behavioral therapy for children (CBT-C). Parental support and guidance. Play therapy and creative expression.	Cognitive behavioral therapy for insomnia (CBT-I). Imagery rehearsal therapy (IRT). Mindfulness and relaxation techniques.
Impact on Daily Functioning [35]	It can affect mood, behavior, and school performance.	Affects productivity, relationships, and quality of life.
Long-term Consequences [27]	It mostly resolves with age, though it may persist in cases of trauma or chronic stress.	It can become chronic in adults, particularly in those with PTSD or mental health issues.

Psychosocial treatment modalities

CBT and IRT are two prominent cognitive-behavioral approaches for treating nightmares. CBT focuses on altering negative thought patterns and behaviors related to nightmares, aiming to help individuals reframe their perceptions of these distressing dreams [36]. This reframing process can reduce the anxiety and distress associated with nightmares. IRT, on the other hand, specifically addresses the content of nightmares by encouraging individuals to visualize a new, positive ending to their distressing dreams while awake. This mental rehearsal can modify the emotional response to the nightmare, reducing frequency and intensity. Research has demonstrated that both CBT and IRT are effective in decreasing nightmare frequency and severity, with IRT showing particularly strong efficacy across various populations, including those with PTSD. The therapeutic benefits of these approaches often persist over time, indicating lasting improvements for those affected by nightmares [37].

Mindfulness practices, originating from Buddhist traditions, also play a significant role in reducing nightmares. Mindfulness involves cultivating present-moment awareness and accepting thoughts and feelings without judgment. Techniques such as mindfulness-based stress reduction (MBSR) and mindfulness-based cognitive therapy (MBCT) can be integrated into treatments for nightmares [38]. These practices promote relaxation and emotional regulation, which can alleviate the anxiety frequently associated with nightmares. Studies suggest that mindfulness can be an effective self-help intervention, particularly for individuals who may be hesitant to seek professional assistance. By fostering a non-judgmental awareness of their experiences, individuals may find it easier to cope with the distress of nightmares, leading to a reduction in their frequency and intensity [39].

Hypnotherapy is another approach that can be used to address nightmares. This technique involves inducing a trance-like state to enhance focus and suggestibility, allowing individuals to explore the underlying causes of their nightmares and reframe their experiences. Although research on the effectiveness of hypnotherapy for nightmares is still developing, preliminary findings suggest it may help reduce the intensity and frequency of nightmares by addressing subconscious fears and anxieties [40]. However, more robust evidence is needed to establish its efficacy compared to other established treatments. The potential of hypnotherapy lies in its ability to facilitate deep relaxation and promote positive imagery, which can benefit individuals struggling with recurrent nightmares [40].

Family therapy and group counseling provide supportive environments for individuals dealing with nightmares. Involving family members in therapy can help address dynamics that may contribute to nightmares, particularly in children. Family therapy can improve communication and support, helping individuals feel more secure and understood [41]. Group interventions allow individuals to share their experiences and coping strategies, reducing feelings of isolation. These settings foster community and offer additional emotional support, enhancing treatment outcomes. By encouraging open dialogue and shared experiences, family and group interventions create a supportive network that promotes healing and resilience [41].

Innovative approaches are being explored to enhance nightmare treatment. Virtual reality (VR) is one such method, creating immersive environments for exposure therapy that allow individuals to confront nightmare themes in a controlled setting. This technology helps desensitize individuals to the fears associated with their nightmares. Similarly, biofeedback techniques teach individuals to control physiological responses linked to anxiety and nightmares, promoting relaxation and emotional regulation [42]. Integrating artificial intelligence (AI) into personalized treatment plans is another exciting development. AI can tailor interventions based on individual responses and preferences, improving treatment effectiveness by adapting to the patient's specific needs. These emerging therapies promise to enhance nightmare management and provide more effective tools for coping with these experiences [42]. The key features and effectiveness of psychosocial treatment modalities for nightmares are summarized in Table 2.

**Table 2:** Psychosocial treatment modalities for nightmares: key features and effectiveness

Treatment Modality	Description	Key Features	Effectiveness
Cognitive Behavioral Therapy (CBT) [43]	A structured therapy aimed at changing negative thought patterns and behaviors associated with nightmares.	Identifies and challenges distorted thoughts. It includes techniques like IRT.	Highly effective for reducing nightmare frequency and intensity in both adults and children.
Imagery Rehearsal Therapy (IRT) [37]	A specific type of CBT is where patients practice changing the endings of their nightmares while awake.	Involves rescripting nightmares with positive endings. Focuses on visualizing alternative outcomes.	Proven effective, especially for post-traumatic stress disorder (PTSD)-related nightmares in adults.
Mindfulness-Based Therapies [39]	Focus on relaxation techniques and increasing present-moment awareness to reduce nightmare intensity.	Techniques include breathing exercises, meditation, and body scanning.	It helps reduce the emotional impact of nightmares and benefits both adults and children.
Hypnotherapy [44]	Uses guided relaxation and focused attention to alter the subconscious and reduce nightmare occurrences.	Helps in reducing anxiety associated with nightmares. It can be combined with other therapeutic modalities.	Effective in decreasing nightmare frequency, though research on long-term effects is still evolving.
Play Therapy (for Children) [45]	Uses play-based activities to help children process emotions and reduce the occurrence of nightmares.	Provides a safe space for children to express fears. Helps in emotional regulation.	Effective for young children, especially when integrated with parental guidance.
Group Therapy [46]	Involves patients sharing experiences and coping strategies in a group setting to alleviate nightmare distress.	Provides peer support and shared learning. Commonly used in combination with other therapies.	Particularly effective for PTSD patients and those experiencing isolation due to nightmares.
Family Therapy [41]	Engages family members to understand and support the individual’s experience with nightmares.	Focuses on improving family dynamics and reducing stress triggers at home.	Effective for children and adults, especially when nightmares are linked to family stress or trauma.
Virtual Reality (Emerging Therapy) [42]	Uses virtual environments to simulate nightmares and practice coping mechanisms in a controlled setting.	Allows patients to confront fears in a safe, controlled manner. Combines with CBT techniques.	Emerging evidence shows potential in treating nightmare-related PTSD in adults, though research is still limited.
Biofeedback (Emerging Therapy) [47]	Uses sensors to monitor physiological stress responses and helps patients control them.	Monitors heart rate, muscle tension, and brain waves. Patients learn to manage stress responses.	Shows promise in reducing nightmare-related anxiety; ongoing research is needed to establish long-term efficacy.

Comparative analysis of psychosocial strategies

Research indicates that psychosocial strategies for treating nightmares exhibit varying effectiveness between adults and children. In adults, CBT, especially IRT, has demonstrated strong efficacy in reducing nightmare frequency and distress [23]. Systematic reviews have highlighted that exposure-based treatments significantly decrease nightmare severity and frequency across various adult populations, including those with PTSD. These therapies often produce rapid results, with many adults experiencing relief within just a few sessions [23]. Conversely, treatment efficacy in children is less well-documented, with fewer high-quality studies available. However, parental involvement and psychoeducation are essential, as they help create supportive environments that can alleviate nightmare-related anxiety [48].

Strategies such as play therapy and family-based interventions may also be beneficial. While some children respond well to these approaches, more clinical trials specifically targeting pediatric populations are needed to determine the most effective strategies for this age group [48]. The effectiveness of treatments for nightmares can differ significantly in terms of short-term and long-term outcomes. Many studies report immediate reductions in nightmare frequency and distress following interventions like IRT and exposure therapy [10]. These treatments often yield quick results, particularly in adults, where patients may experience relief within a few sessions. For example, adults undergoing IRT frequently report a marked decrease in the intensity and occurrence of their nightmares shortly after beginning therapy [10].

However, longitudinal studies suggest that while short-term improvements are common, the sustainability of these effects can vary. For adults, continued practice of techniques learned in therapy can help maintain benefits over time. In children, long-term outcomes are less clear, with some studies indicating that nightmares may persist or recur if underlying psychological issues are not adequately addressed. Thus, ongoing support and follow-up may be necessary to ensure lasting improvements in both age groups, underscoring the importance of a comprehensive approach that includes both immediate intervention and long-term care [49].

Cultural and socioeconomic factors significantly influence the adaptation and effectiveness of nightmare therapies. Different cultural backgrounds may shape the perception and interpretation of nightmares. For instance, in some cultures, nightmares might be viewed as spiritual messages or omens, affecting how individuals respond to treatment. Therapies may need to be tailored to respect these cultural beliefs while addressing the psychological aspects of nightmares. Cultural sensitivity can enhance the therapeutic alliance and improve treatment adherence [50]. Socioeconomic status also impacts access to treatment and the type of support available. Families with lower socioeconomic status may face additional stressors exacerbating nightmares in children, such as unstable living conditions or parental mental health issues [51]. Therefore, interventions must consider these factors, potentially incorporating community resources and support systems to enhance treatment effectiveness for diverse populations. By addressing cultural and socioeconomic considerations, therapists can better tailor their approaches to meet individual needs, ultimately leading to improved outcomes in nightmare treatment [51].

Challenges and future directions

Despite the availability of effective treatments for nightmares, several barriers can impede access to care and successful management of the condition. One major challenge is the stigma associated with seeking help for mental health issues. Many individuals may feel embarrassed or ashamed to discuss their nightmares, fearing judgment or misunderstanding from others. This stigma can prevent them from seeking the support they need. Additionally, access to care remains a critical issue, particularly in rural or underserved areas where mental health professionals are scarce [52]. Limited availability of specialists can result in long wait times or inadequate treatment options for those suffering from persistent nightmares. Furthermore, a lack of awareness about the treatability of nightmares can lead individuals to believe they must simply endure their distressing dreams without seeking help. Many may not recognize that persistent nightmares are a legitimate mental health concern that warrants professional intervention [52].

While significant progress has been made in understanding and treating nightmares, several areas require further investigation. One such area is the impact of digital media on the prevalence and characteristics of nightmares. With the increasing use of digital devices and exposure to disturbing content, it is essential to study how these factors influence the frequency and nature of nightmares in both children and adults. Additionally, more research is needed to explore cross-cultural differences in the experience and interpretation of nightmares [19]. Understanding how cultural factors shape the perception of nightmares can help tailor treatment approaches to meet the needs of diverse populations better. Longitudinal studies are also necessary to assess nightmare treatments' long-term effectiveness and impact on overall mental health and well-being. Addressing these research gaps will contribute to a more comprehensive understanding of nightmares and their treatment [19].

Several promising trends may shape the future of nightmare treatment as research advances. One significant trend is the integration of technology into therapeutic practices. Innovations such as virtual reality and mobile applications may enhance the delivery and accessibility of nightmare treatments, particularly for individuals facing barriers to in-person therapy [53]. These technologies can offer immersive experiences that help individuals confront and reprocess their nightmares in a controlled environment. Another trend is the potential for interdisciplinary approaches, where collaborations between mental health professionals, sleep specialists, and other healthcare providers lead to more comprehensive and effective treatment strategies [53].

By combining expertise from various fields, practitioners can develop holistic treatment plans that address the multifaceted nature of nightmares. Lastly, personalized medicine may become more prevalent as our understanding of the genetic and biological factors contributing to nightmares improves. Tailoring treatment approaches to individual needs and characteristics could enhance the effectiveness of interventions and improve outcomes for those affected by persistent nightmares [53]. Challenges and future directions in treating nightmares are summarized in Table 3.

**Table 3:** Challenges and future directions in the treatment of nightmares

Category	Challenges	Future Directions
Access to Care [54]	Limited access to mental health professionals, especially in rural or underserved areas.	Expand telemedicine and online therapy platforms to reach a wider population.
Stigma [55]	The social stigma surrounding seeking psychological help, particularly for nightmares, is linked to trauma.	Increase awareness and education to reduce stigma and normalize mental health care.
Lack of Awareness [56]	Limited public knowledge of available psychosocial treatments for nightmares.	Promote public health campaigns and integrate mental health education in schools and communities.
Cultural Barriers [57]	Cultural differences may impact the acceptance and effectiveness of certain therapies.	Adapt treatment modalities to be culturally sensitive and inclusive.
Research Gaps [58]	Lack of comprehensive, long-term studies on the effectiveness of psychosocial interventions.	Conduct more longitudinal studies and randomized controlled trials to validate and refine treatment strategies.
Treatment Personalization [59]	A one-size-fits-all approach may not be effective for diverse populations.	Develop personalized treatment plans using artificial intelligence (AI) and machine learning for more individualized care.
Technological Integration [60]	Limited use of emerging technologies such as virtual reality (VR) and biofeedback in mainstream therapy.	Invest in research and development of innovative technologies like virtual reality and biofeedback for nightmares.
Training and Resources [61]	Insufficient training for mental health professionals on specific therapies like imagery rehearsal therapy (IRT) and hypnotherapy.	Expand professional training programs and resources to equip practitioners with advanced treatment modalities.
Barriers in Pediatric Care [62]	Difficulty in applying adult-centric treatments to children.	Tailor therapies like cognitive behavioral therapy (CBT) and play therapy for younger age groups with age-appropriate approaches.
Long-Term Efficacy [63]	Uncertainty about the long-term success of some psychosocial strategies.	Focus on follow-up studies to assess long-term benefits and side effects of psychosocial treatments.
Interdisciplinary Approaches [64]	Lack of collaboration between psychologists, psychiatrists, and sleep specialists.	Promote interdisciplinary approaches combining psychological, medical, and technological interventions.

## Conclusions

Nightmares are more than just disturbing dreams; they are significant psychological events that can profoundly impact an individual’s mental and physical well-being. Their prevalence, especially among vulnerable populations such as children and those with underlying mental health conditions, underscores the necessity of addressing them with effective treatment strategies. While pharmacological options exist, psychosocial approaches such as CBT, IRT, and mindfulness-based interventions offer a holistic and often more sustainable solution. These strategies not only reduce the frequency and intensity of nightmares but also empower individuals by addressing the underlying cognitive and emotional factors contributing to their distress. By integrating these non-pharmacological interventions into treatment plans, healthcare providers can enhance the quality of life for those affected by nightmares, helping them achieve more restful sleep and better overall mental health.
